# GSK3β: a plausible mechanism of cognitive and hippocampal changes induced by erythropoietin treatment in mood disorders?

**DOI:** 10.1038/s41398-018-0270-z

**Published:** 2018-10-11

**Authors:** Becky Inkster, Gwyneth Zai, Gemma Lewis, Kamilla W. Miskowiak

**Affiliations:** 1 Wolfson College, University of Cambridge, Cambridge, UK; 2 Department of Psychiatry, University of Cambridge, Cambridge, UK; 3 Cambridgeshire & Peterborough NHS Foundation Trust, Cambridge, UK; 4 Behavioural and Clinical Neuroscience Institute, University of Cambridge, Cambridge, UK; 5 Neurogenetics Section, Molecular Brain Science Department, Campbell Family Mental Health Research Institute, and Mood & Anxiety Division, Centre for Addiction and Mental Health, Toronto, Canada; 6 Department of Psychiatry, University of Toronto, Toronto, Canada; 7 Psychiatric Epidemiology at the Division of Psychiatry, University College London, London, UK; 8 Psychiatric Centre Copenhagen, Copenhagen University Hospital, Rigshospitalet, København, Denmark

## Abstract

Mood disorders are associated with significant psychosocial and occupational disability. It is estimated that major depressive disorder (MDD) will become the second leading cause of disability worldwide by 2020. Existing pharmacological and psychological treatments are limited for targeting cognitive dysfunctions in mood disorders. However, growing evidence from human and animal studies has shown that treatment with erythropoietin (EPO) can improve cognitive function. A recent study involving EPO-treated patients with mood disorders showed that the neural basis for their cognitive improvements appeared to involve an increase in hippocampal volume. Molecular mechanisms underlying hippocampal changes have been proposed, including the activation of anti-apoptotic, antioxidant, pro-survival and anti-inflammatory signalling pathways. The aim of this review is to describe the potential importance of glycogen synthase kinase 3-beta (GSK3β) as a multi-potent molecular mechanism of EPO-induced hippocampal volume change in mood disorder patients. We first examine published associations between EPO administration, mood disorders, cognition and hippocampal volume. We then highlight evidence suggesting that GSK3β influences hippocampal volume in MDD patients, and how this could assist with targeting more precise treatments particularly for cognitive deficits in patients with mood disorders. We conclude by suggesting how this developing area of research can be further advanced, such as using pharmacogenetic studies of EPO treatment in patients with mood disorders.

## Mood disorders and cognitive deficits

Mood disorders affect ∼20% of the general population^1^ and for individuals suffering from a mood disorder, there is a 5–6% lifetime risk of completed suicide^2^. Major depressive disorder (MDD) is ranked as the third most prevalent condition associated with disability^3^ and is estimated to be the second leading cause of disability worldwide by 2020^4^. Bipolar disorder (BD) is also on the top ten list of most debilitating mental illnesses^3^ and is associated with significant psychosocial and occupational disability^5^. Both mood disorders, MDD and BD, are debilitating and chronic psychiatric disorders that cause significant suffering and burden in individuals with these illnesses and their families and friends, as well as reducing their quality of life^6–8^.

Treatment of MDD and BD has focused on reducing mood symptoms;^9^ however, cognitive deficits are a core symptom domain of mood disorders^10^ that prolongs illness duration and reduces the likelihood of recovery^11,12^. Cognitive dysfunction also contributes to socio-occupational impairment^13,14^, which represents the largest economic cost of mood disorders for society^15,16^. Patients with MDD have consistently displayed difficulties in attention (e.g., in effortful attention, as well as automatic processing), declarative memory (e.g., verbal learning and memory, visuospatial learning and memory and episodic memory), and executive function (e.g., response inhibition, problem solving and planning, verbal fluency, decision-making and mental flexibility)^17^. These deficits are particularly pronounced in response to information that is emotionally or socially relevant. Similar but more severe deficits, specifically in verbal learning, spatial working memory, set-shifting and sustained attention, have been reported in patients with BD^18,19^. While neurobiological mechanisms of cognitive impairments in mood disorders are unclear, converging preclinical, human neuroimaging and post-mortem evidence suggest that they may arise from disrupted neuroplasticity and associated structural changes in hippocampal volume^20–22^. This highlights the potential of novel treatments with direct and lasting effects on neuroplasticity changes to induce enduring structural alterations and effectively alleviate cognitive deficits.

Pharmacological treatments for mood disorders have limited effects on cognitive dysfunction^23,24^ and are, in some cases, associated with adverse effects on cognition due to anticholinergic, sedative, extrapyramidal and/or blunting effects^25^, which may exacerbate patients’ persistent cognitive impairments during periods of remission (i.e., when patients are relatively symptom-free)^26^. Existing cognitive enhancing drugs (i.e., medications aiming to improve cognitive functions) have shown limited pro-cognitive effects in depressed patients^27^. Among the most promising cognition treatments are vortioxetine, which has shown replicated effects on psychomotor speed in symptomatic MDD^28^, modafinil that improved some aspects of cognition in a study of remitted MDD^29^, transcranial direct current stimulation that improved working memory in symptomatic MDD^30,31^, lurasidone that improved a global measure of cognition in remitted BD^32^ and erythropoietin (EPO) that improved several cognitive domains in symptomatic MDD and remitted BD^33,34^. However, despite these promising findings, there are no clinically available effective treatments for cognitive impairment in mood disorders to date^35,36^. Indeed, many studies have examined the efficacy of existing and novel interventions to reduce cognitive dysfunction in patients with mood disorder;^35,36^ however, cognition trials in this area have faced some important methodological challenges that may negate the interpretations and significance of findings^36,37^. Although preliminary evidence showed promising effects of psychological interventions for cognitive dysfunction, such as cognitive remediation in patients with MDD^33,38^, we recently demonstrated a lack of beneficial effects of this intervention for BD patients in a randomized, controlled clinical trial^39^. Notably, this trial was limited by a small sample size (*n* = 44), short follow-up times (12 weeks) and lack of enrichment for the primary outcome (objectively-assessed verbal memory dysfunction). Indeed, emerging evidence indicates that cognitive remediation programs may be useful in BD and there are several ongoing cognitive remediation trials in BD.

Recent randomized, placebo-controlled trials demonstrated that 8 weekly doses of erythropoietin (EPO) reduced cognitive dysfunction in patients with treatment-resistant depression (TRD)^33^ and in patients with BD in partial remission^34^. Treatment-resistant depression was defined as lack of remission after ≥ 2 adequate antidepressant treatments with 2 different classes of antidepressant drugs in previous or current depressive episodes^33^. The improvement of verbal memory after EPO vs. saline treatment across TRD patients and BD patients was of a moderate effect size (change in RAVLT total score, mean [SD]: EPO: 6.4 [8.8]; saline: 2.1 [8.0]; *d* = 0.54). Structural magnetic resonance imaging (MRI) assessments of patients from these two trials revealed that memory improvement was associated with normalization of volume loss in a subfield of the left hippocampus corresponding to the cornu ammonis 1–3 (CA1–3) and subicilum^40^. Post hoc exploratory assessments of the mean surface displacement values revealed that the subfield hippocampal volume change was of a large effect size (hippocampal surface displacement, mean [SD]: EPO: 0.04 [0.08]; saline: −0.05 [1.0]; *d* = 0.90). However, the biological mechanisms linking EPO to increased hippocampal volume in mood disorders remain unknown.

## EPO biology

EPO is a glycoprotein hormone cytokine that plays important roles in regulating red blood cell synthesis (i.e., hematopoiesis)^41^, trafficking of immune cells, anti-apoptotic actions, neurodevelopment^42^, neuroprotection and cognitive function^43,44^. EPO and its receptor are expressed in multiple organ systems and have been shown to interact closely with the nervous, vascular, immune and reproductive systems^45–47^. EPO is produced and secreted predominantly in the kidney, but it is also expressed in brain regions including the hippocampus, amygdala, temporal cortex, prefrontal cortex, internal capsule and midbrain^45,48,49^ as well as the liver and the uterus^47^. Expression of EPO and its receptor have also been found in neurons, glial cells, endothelial cells and adult neural progenitor cells. Expression levels are high during human embryonic brain development, but remain present in adulthood^45^. EPO functions in a hypoxia-sensitive manner meaning that stimuli such as hypoxia and stress (i.e., cellular changes such as hypoglycaemia, electrolyte imbalance, anaemia, infections and loss of endogenous anti-oxidants, etc.) can affect EPO and its receptor^45–47^, which can have pleiotropic effects in the modulation of apoptotic and immune activities^50^ as well as neurotrophic and neuroprotection effects^46^. Specifically, hypoxia-inducible factor (HIF) rapidly upregulates the expression of the EPO receptor, EPO-R, in cells of the Central Nervous System (CNS) and of EPO synthesis by neurons and astrocytes^45^. Extracellular EPO then binds to EPO-R on the cell membrane, which triggers the intracellular JAK2 (janus kinase 2) signalling. This results in the activation of several signal transduction pathways including STAT5 (signal transducer and activator of transcription 5), PI3K (phosphatidylinositol 3-kinase)/Akt (protein kinase B), NFκB (nuclear factor-κB) and MAPK (mitogen-activated protein kinase). These pathways switch on signalling cascades that lead to long-lasting biological protective and reparative responses, which may be important for future treatment of cognitive impairments in neuropsychiatric disorders including depression^46^. Specifically, relevant down-steam effects of these signalling cascades include activation of anti-apoptotic, antioxidant and anti-inflammatory signalling in neurons, glial and cerebrovascular endothelial cells, and promotion of dendritic sprouting, neurogenesis, hippocampal brain-derived neurotrophic factor (BDNF) and long-term potentiation^51–53^. Erythropoietin was also shown to exert neuroprotective effects by inhibiting the activity of the enzyme glycogen synthase kinase 3-beta (GSK3β)^54,55^, as will be discussed in greater detail later in this review. This may be particularly relevant in relation to mood disorders since GSK3β is a key activator of cell death and other functions involved in mood disorders, hippocampal volume, glucocorticoid regulation and neuroplasticity^56–58^.

It was a conceptual break-through that systemic administration of high-dose (> 500 International Units [IU]/kg) EPO was shown to cross the blood-brain barrier (BBB)^49^ and facilitate neuroprotection and neuroplasticity in animal models of neurodegenerative and neuropsychiatric conditions^59^ in addition to after acute neural injury^60–62^. While it is unclear whether EPO crosses the BBB via an active transport mechanism or in an unspecific manner, it is evident that systemically administered high-dose EPO enters the brain to an extent that is sufficient for neuroprotection (ibid.). Accordingly, administration of such high doses of EPO to humans (through injections of 40,000–48,000 IU/ml)^33,34,63–65^ improved brain function and cognition after short-term (1 week) and longer-term (8–12 weeks) treatment. In contrast, short-term administration (3 days) administration of lower-dose EPO (30,000 IU to men of 74 ± 7 kg [mean ± SD]; corresponding to < 500 IU/kg) produced no cognitive benefits in healthy men^66^ and 12 weeks low-dose EPO treatment (8000 IU/ml) produced no neural or cognitive benefits with schizophrenia^63,67^. Although no more precise pharmacokinetic or pharmacodynamic studies have been performed, this evidence indicates that high doses of EPO are required for neuroprotection and cognitive enhancement.

EPO has also been used to treat anaemia, ischaemia and reperfusion injuries (i.e., stroke, heart attack)^68^, neurological disorders (i.e., seizures^69^, spinal cord ischaemia, Alzheimer’s disease, Parkinson’s disease and demyelinating disease^47^), and retinal disease^47^ and neuropsychiatric disorders^33,34,46^. Thus, knowledge of the underlying mechanisms of EPO may provide important insights for future therapeutic strategies for the treatment of neuropsychiatric, neurodegenerative, inflammatory and autoimmune-related disorders.

**Fig. 1:**
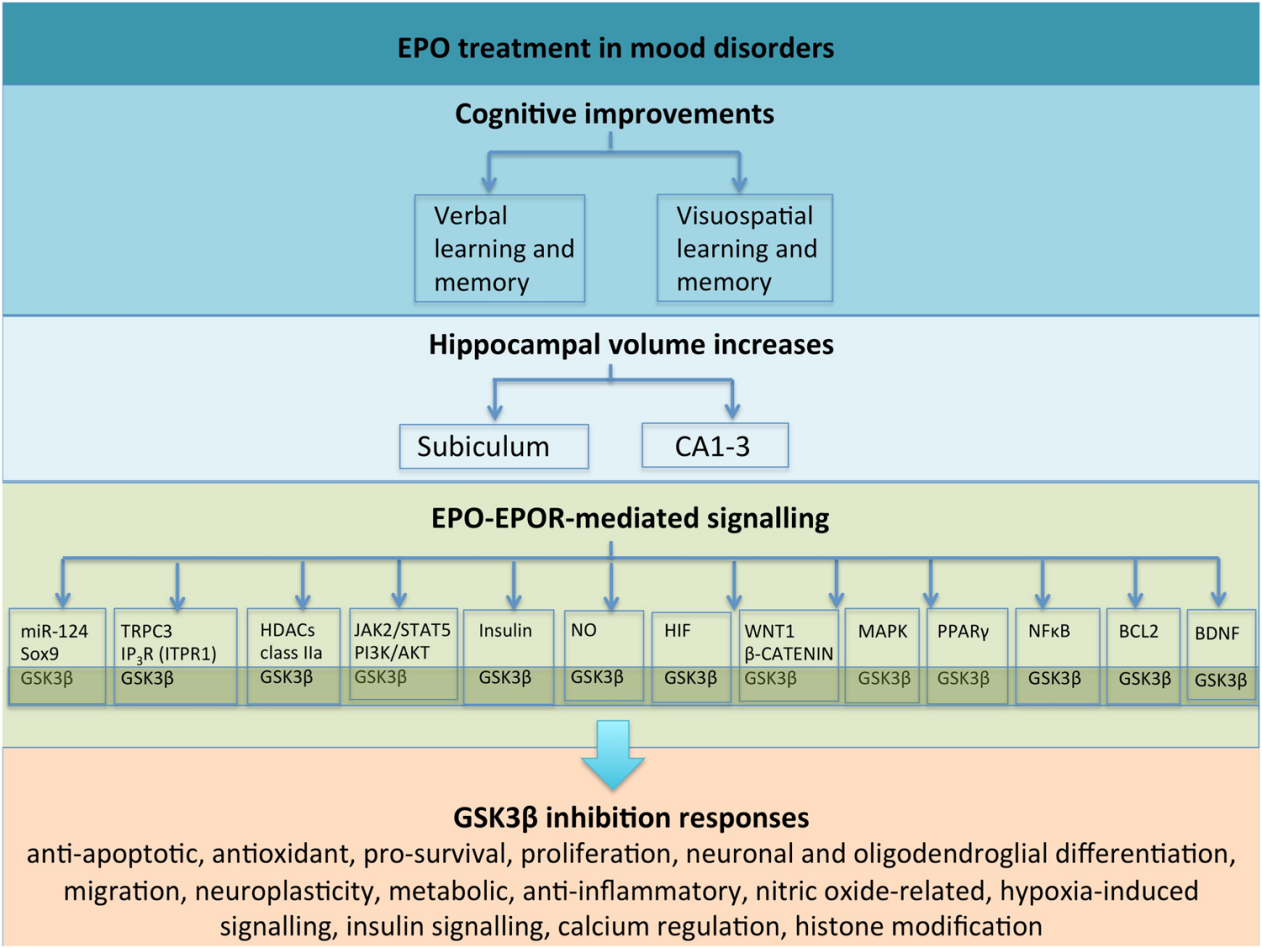
This overview schematic summarizes the complex, interrelated relationships between EPO treatment in mood disorders, cognitive deficits, hippocampal changes and EPO’s potential mechanisms of action through the GSK3β inhibition . Notably, complex relationships exist across signalling pathways and molecules, which have not been illustrated

In this review, we highlight evidence collectively suggesting that inhibition of GSK3β acts as a multi-potent molecular mechanism that may mediate multi-potent effects of EPO on hippocampal volume changes in depression (Fig. 1). Understanding the complex relationship between EPO and GSK3β (and its pleiotropic regulatory role across its large genetic network) on cognitive functioning in depressed patients may help reveal new drug targets (both upstream and downstream), aid precision medicine, and ultimately reduce disability and mortality for mood disorders.

## Narrative review search methodology

The following search terms were included in this review: cognition, cognitive functions or dysfunction or impairment or deficits, cognitive enhancers or enhancement, mood disorders, depression, bipolar disorder (BD), major depressive disorder (MDD), treatment-resistant depression (TRD), erythropoiten (EPO), glycogen synthase kinase-3 beta (*GSK3β*), hippocampus, hippocampal volume or structure, molecular pathway and biology or biological. Several search engines were used, including PubMed and Medline. This review has mainly focused on unipolar and bipolar depression and therefore, only the most recent reviews on other disorders such as neurological and cardiac diseases have been included for references. Two factors led us to choosing a narrative style for the review paper: firstly, to our knowledge, this is the first review paper to bridge these complex interrelated topics in the literature and, secondly, it was not our intention to perform an extensive systematic search for each of the topics independently as this would be an enormous undertaking beyond the scope of our narrative approach.

## EPO treatment and cognitive function

Studies in patients with schizophrenia, and multiple sclerosis, have shown that 8–12 weeks of high-dose (40,000–48,000 IU) EPO treatment improves cognitive functioning that lasts for up to 6 months after treatment completion, long beyond red blood cell normalization^63,64^. This indicates that the pro-cognitive effects of EPO are not directly related to changes in the vascular system. Indeed, the effects of EPO on neurocognitive function in humans seem to be mediated through neurobiological actions rather than indirect increases in red blood cells^65,70^. In particular, these studies demonstrated that a single high dose of EPO (40,000 IU) versus saline improves neural and cognitive measures of memory and executive functioning in healthy volunteers without affecting red blood cells (ibid.). Based on this evidence, Miskowiak et al.^33,34^ conducted a randomised, placebo-controlled clinical trial examining the effects of 8 weekly infusions of EPO (40,000 IU) on mood symptoms and cognitive dysfunction in patients with TRD and patients with BD in partial remission. EPO treatment improved verbal memory in TRD patients and speed of complex cognitive processing across attention, memory and executive function in BD patients relative to placebo treatment. These cognitive changes were independent of changes in mood symptoms and were maintained several weeks after red blood cell normalisation at a 6-week follow-up at which time EPO-treated patients displayed structural increase in the left hippocampus^40^ and changes in task-related neural activity within a fronto-parietal network^71,72^. Importantly, post hoc analyses showed that the structural hippocampal increase and task-related neural activity change correlated with the observed improvements in EPO-treated patients’ cognitive functions, whereas no influence was found of changes in red blood cells, mood symptoms, diagnosis, age or gender^40,71,72^.

Effects of EPO have also been demonstrated on neural and cognitive responses to facial expressions in healthy volunteers^70,73^ and were subsequently replicated in a sample of patients with acute depression^74^. Long-term EPO treatment did not improve the primary measure of depression severity in an 8-week trial (Hamilton Depression Rating Scale [HDRS] score), but this may be a result of suboptimal statistical power^75^ and the use of HDRS, which might underestimate other less relevant depressive symptom domain and burden of illness that correlate poorly with depression severity;^76^ however, improvement in several other depression-relevant outcomes including self-rated depression and quality of life were observed, suggesting that further investigations of the antidepressant efficacy of EPO in larger-scale trials are warranted^33^. Given this evidence demonstrating the potential impact of EPO on cognitive function and mood symptoms, it is important to elucidate the biological mechanisms underlying alterations of neural processing.

## GSK3β: biological mechanism of mood disorders

GSK3β is a highly active proline-directed serine-threonine protein kinase. It contributes to diverse cellular functions including gene expression, neurogenesis, neuroplasticity, cell survival, differentiation, migration, stress responses, cell structure, cell death, the immune system, neurotransmitter systems, metabolism and other functions^77–80^. GSK3β inhibitors increase proliferation, migration and differentiation of neural stem cells in the adult hippocampal dentate gyrus^81^. GSK3β is ubiquitously expressed throughout the brain, most prominently in the cerebral cortex and hippocampus (Allen Brain Atlas). GSK3β is a particularly unique protein kinase^82^ that can be inactivated through the action of various kinases, such as Akt/protein kinase B, protein kinase A and protein kinase C on the ninth position of serine (Ser9)^83^.

Several neurogenetics studies have investigated associations between *GSK3β* and mood disorders. The GSK3β gene (*GSK3β*; OMIM 605004) was mapped to chromosome 3q13.3^84^. Functional single nucleotide polymorphisms (SNPs) have been identified in *GSK3β*; for example, a promoter T to C polymorphism at position −50 (rs334558) with the T allele having a higher in vitro transcriptional activity and an intron 5T to C polymorphism at position −157 (rs6438552) with the T allele lacking exons 9 and 11 and has been associated with an increased level of GSK3β^85^. Several studies have investigated genetic variants in *GSK3β* as risk factors for MDD^86,87^ and BD^88^. Other studies have focused on anxiety symptoms in MDD and P300 waveform^89^, psychotic symptoms in MDD and BD^90^, age of onset in MDD^91^ and BD^92^, suicidal behaviour in MDD^93^ and combined cases of MDD and schizophrenia patients^94^. Furthermore, *GSK3β* polymorphisms have been examined as a predictor of antidepressant response^95^ and lithium response^96,97^.

Neuroimaging genetic studies of mood disorders have reported associations between *GSK3*β variation and hippocampal volume. A genetic association study of numerous *GSK3β* SNPs and brain-wide grey matter volume using MRI-based voxel-based morphometry was conducted in a sample of 134 patients with recurrent MDD and 144 healthy controls^56^. Disease modulated associations were reported between grey matter volume in the right hippocampus and bilateral temporal cortex and a functional intronic *GSK3β* polymorphism, rs6438552. The same direction of association was observed in a larger, independent sample of healthy volunteers between the same *GSK3β* polymorphism and hippocampal volume using different neuroimaging methods^98^. This polymorphism has also been associated with altered resting state networks in MDD patients^99^. Based on in vitro work, this polymorphism alters the splice acceptor site leading to exclusion of exons 9 and 11, which alters the protein’s function to then hyperphosphorylate the substrate, microtubule-associated protein tau^85^. Further in vivo and in vitro work is required to understand how this modified GSK3β protein regulates other substrates. Additional associations between hippocampal volume and genetic variation involving GSK3β-related pathways and other directly interacting proteins have also been reported^57,58^.

## Identifying putative connections between GSK3β, erythropoietin, hippocampus, cognition and mood disorders

The hippocampus is an important brain region implicated in mood disorders. Specifically, changes in the neural circuitry of the hippocampus have been implicated in cognitive deficits in patients with mood disorders^100–102^, which may arise in part from the disruption of neuroplasticity^67^. Disturbance in hippocampal neuroplasticity has been hypothesised to play an aetiological role in mood disorders and may result from chronic inflammatory processes and over-activation of stress responses^103–105^. This is consistent with evidence showing that stress-induced glucocorticoid production is associated with reduced hippocampal neurogenesis, hippocampal memory deficits and depression-like behaviour in animals^106–109^. Moreover, a recent meta-analysis^110^ supported an overall significant hippocampal volume reduction in patients with MDD relative to controls and several additional studies reported hippocampal subiculum shape abnormalities in patients with depression^111–113^.

The involvement of GSK3β in EPO-mediated neuroprotection via PI3K/AKT is well documented in the literature (e.g. see refs ^45,114–116^). In the context of primary hippocampal neurons, EPO treatment triggers pro-survival mechanisms by activation of PI3K/AKT^45^, which suppresses downstream target GSK3β (i.e., by increasing phosphorylation of Ser9 in GSK3β)^117^. In contrast, PI3K/AKT pathway inactivation results in GSK3β pro-apoptotic functions. In a recent study, Ma and colleagues^116^ administered exogenous EPO to rats for 4 weeks using an animal model of vascular dementia. Their results indicated improvements in memory impairment, promotion of hippocampal dendritic spine growth as well as deactivation of GSK3β via an EPO-R/JAK2/STAT5/PI3K/Akt/GSK3β pathway^116^ (Fig. 1).

Another mechanism of action of EPO treatment that could be linked with GSK3β function is through the central role that GSK3β plays in neuronal and oligodendroglial differentiation. A recent study by Hassouna and colleagues^118^ examined the effects of EPO in young, healthy mice administered EPO for 3 weeks. The authors reported an approximately 20% increase in hippocampal CA1/CA3 neurons and oligodendrocytes, and they detected a significant enhancement of neuronal and oligodendroglial differentiation rather than proliferation^118^. Using neural stem cells and hippocampal cultures, the authors found that EPO administration decreased the transcription factor Sry-box 9 (Sox9) and increased micro RNA 124 (miR-124). miR-124 is known to regulate Sox9 function and drive neuronal differentiation^118^. We highlight evidence showing an interconnected relationship between GSK3β, Sox9 and miR-124. Sox9 interacts with GSK3β via its targets in the Wnt signalling pathway^119,120^. For example, Sox9 inhibits the GSK3β-dependent Wnt/beta-catenin signaling pathway in chondrocyte differentiation by promoting beta-catenin phosphorylation in the nucleus. This finding is in keeping Hassouna and colleagues^118^ in that EPO inhibits Sox9 although it should be noted that different tissues and models were used and so further investigations are warranted for mood disorders. Other Sox-related genes should also be explored given that, for example, Sox17 regulates the Wnt/β-catenin signaling pathway via GSK3β in oligodendrocyte progenitor cells^121^. Furthermore, miR-124 co-regulates neuronal differentiation and dendritic architecture via the AKT/GSK3β-dependent pathway^122^. Its regulation of GSK3β hippocampal expression may have implications for chronic stress and mood disorder pathophysiology^123,124^. Further evidence has shown that miR-124 regulates HDAC4 and GSK3β expression in the hippocampus, which may have important implications for chronic stress and depression^124^ and another study identified associations between *HDAC4* genetic variation and reduced hippocampal volume in two independent MDD cohorts^58^. Notably, another class IIa histone deacetylases (HDAC5) has been implicated in the therapeutic action of EPO whereby researchers found that EPO regulates phosphorylation at two different sites stimulating nuclear export of HDAC5 in rat hippocampal neurons^125^. Collectively and indirectly, these diverse studies provide a plausible link between EPO treatment and its downstream effects on GSK3β function (Fig. 1), which require much greater examination in order to delineate specific and selective effects.

Research has shown that EPO stimulates calcium influx. In terms of biological mechanisms, one study demonstrated that interactions between inositol 1,4,5-trisphosphate (ITPR1; alias, IP_3_R) and transient receptor potential cation channel subfamily C member 3 (TRPC3) is required for epo-modulated Ca^2+^ influx, which was reduced under conditions of mutated or deleted IP_3_R binding sites on TRPC3^126^. *ITPR1* (alias, IP_3_R) genetic variation was recently associated with reduced hippocampal volume in two independent MDD cohorts, which lead the authors to speculate that mood disorders, and specifically cognitive changes, may involve mechanisms related to ITPR, endoplasmic reticulum (ER) stress, the unfolded protein response (UPR) system and GSK3β signalling^56^.

Another possible way in which EPO treatment could be linked with GSK3β function is through anti-apoptotic mechanisms. Several biological models have implicated GSK3β as a key activator of cell death^79^ and so inactivation of GSK3β may therefore promote cell viability. For example, evidence has demonstrated a molecular relationship between EPO, GSK3β and the mitochondrial cell death pathway; EPO suppresses 6-hydroxydopamine (6-OHDA)-induced apoptosis by increasing phosphorylation of Ser9 in GSK3β (i.e., increasing GSK3β inhibition)^54^. Neuroprotective effects against apoptosis were observed for both EPO and the GSK3B inhibitor 4-benzyl-2-methyl-1, 2,4-thiadiazolidine-3, 5-dione (TDZD8). In contrast, 6-OHDA decreased phosphorylation of Ser9 in GSK3β (i.e., increased GSK3β activity). In this study, decreases in mitochondrial expression of the anti-apoptotic gene B-cell lymphoma 2 (*Bcl-2*) were also observed (Fig. 1). Other related work has also described a relationship between EPO treatment, increased phosphorylation of Ser9 in GSK3β, and oxidant stress-induced apoptosis^127,128^. Further investigation is crucial to understand how EPO treatment interacts with GSK3β function in different brain tissue types, cellular environments and diseases.

An additional relationship between EPO and GSK3β involves the downstream increase in hippocampal brain-derived neurotrophic factor (BDNF) expression, neurite growth and spine density^53,129^ (Fig. 1). BDNF is highly involved in neuroplasticity, cell survival, differentiation and cell death^130,131^ as well as learning and memory^132–134^. Evidence has shown that GSK3β interacts with BDNF at the protein level; GSK3β overexpression inhibits BDNF-induced cAMP response element-binding (CREB) phosphorylation^135,136^. *GSK3β* and *BDNF* genotype combinations have been associated with MDD^86^.

A complex relationship between EPO, GSK3β, the hippocampus and depression may exist, in part, through nitric oxide (NO)-related pathways. In brief, increased GSK3β mRNA expression was found in post-mortem hippocampal samples from MDD patients, which is consistent with previous animal studies of depression. GSK3β mRNA expression was also significantly correlated with nitric oxide synthase 1 (NOS1) in these same patients^137^, which is in keeping with previous evidence suggesting that nitric oxide activates GSK-3β. EPO can influence oxygen delivery through stimulation of NO production^45^, which may contribute to its neuroprotective role; however, this relationship is complex and very much dependant on the cell and tissue type, and different dose-time exposure conditions (i.e., short-term versus long-term exposure, hypoxia versus normoxia conditions etc.). Possible relationships between EPO, GSK-3β, NOS and hypoxia may exist, although specific mechanisms remain unclear. Hypoxia modulates NOS mRNA and protein levels under specific conditions^138^, GSK-3β overexpression is associated with reduced hypoxia-inducible transcription factor 1α (HIF-1α)^139^, while EPO-R expression is rapidly upregulated by HIF^45^. More detailed work in this area is needed to understand the isoform-specific interactions however (e.g., the role of HIF-1α versus HIF-2α etc.).

GSK3β acts centrally in the canonical Wnt signalling pathway, which is essential for regulating neurodevelopment as well as synaptic maintenance and plasticity in the adult brain^140^. Independent evidence has implicated the Wnt pathway in mood disorders^57,141^. Biological interactions between EPO and the canonical Wnt signalling pathway have been observed in elevated D-glucose models of diabetes^142^. These authors^142^ found that EPO triggered anti-apoptotic responses via the modulation of Wnt1 protein expression that subsequently promoted beta-catenin translocation. The authors^142^ also reported that Wnt1 gene silencing and Wnt1 antagonist administration prevented the protective EPO treatment. Notably, biological interactions between EPO and Wnt signalling have also been a proposed mechanism of action for neurodegenerative diseases^47^.

Peroxisome proliferator-activated receptor-gamma co-factor 1A (*PPARGC1A*) is involved in the PPAR-γ system, which interacts with numerous pathways including the Wnt signalling pathway^57^. Evidence has shown that *PPARGC1A* genetic variation is associated with altered brain volume in MDD patients^57^. Activation of the PPAR-γ system has been shown to improve depressive-like behaviours^105^. It has been proposed that PPAR-γ plays a protective role against ER stress^105^ and that PPAR-γ pro-survival activity is inhibited by HDAC4 activation^143^. Furthermore, the PPAR-γ system has been linked to EPO function^144^. For example, a study examining the therapeutic implications of EPO in type 2 diabetes and insulin resistance found that EPO regulates the PI3K/AKT signalling pathway via PPARγ-dependent activation^144^ (Fig. 1).

Insulin signalling pathways also share complex relationships with both EPO and GSK3β. Evidence has shown that insulin-like growth factor leads to increased EPO and EPOR expression in neuronal cells^46^ and that GSK3β is inhibited by insulin-mediated mechanisms^145^. It has been proposed that impaired insulin receptor-mediated regulation of GSK3β activity is involved with the cognition and depression^146^.

## EPO, GSK3β and pharmacological treatments

Evidence from animal studies suggests that inhibition of GSK3β is a potential mechanism contributing to the antidepressant-like effects of lithium, ketamine^147^ and valproate^148^. Lithium is considered to be the gold standard pharmacological treatment for BD and has pleiotropic effects on multiple cellular systems and pathways^149^. Additionally, lithium treatment results in significant inhibition of GSK3 activity^150,151^, which has been shown to mediate neuroprotective, anti-oxidative and neurotransmission mechanisms. The effect of lithium-induced GSK3 inhibition has also previously been shown to reduce tauopathy and neurodegeneration^152^, and another study demonstrated that lithium (Li^+^) inhibits GSK3 by competition for magnesium (Mg^2+^)^153^. With regard to ketamine, while the literature is inconclusive, there is an indication that ketamine may be effective at treating depression^154^, in particular severe depression, TRD and acute suicidality. With its fast-acting properties^155^, ketamine has been shown to interact with EPO^156,157^. The combination of EPO and ketamine may offer new areas of investigation for mood disorder treatments. The antidepressant actions of ketamine involve GSK3β inhibition^147^. Lithium and other selective GSK3β inhibitors enhance the effects of low doses of ketamine^158^ and the authors suggested that GSK3β activation is an underlying mechanism related to ketamine-induced apoptosis. Low-dose interactions may be of particular interest for reducing the risk of side effects and possible misuse given prior evidence implicating ketamine with misuse and addiction^159^. Ketamine has been shown to modulate inflammatory responses^160^. Acute or chronic use of ketamine has been found to induce cognitive impairments with hyperphosphorylation of tau and apoptosis^161^, and transient behavioural changes similar to schizophrenia (i.e., motor and social behavioural disturbances)^162,163^. However, studies have previously shown that ketamine has anti-inflammatory effects under inflammatory conditions and has been used in surgical procedures in patients with sepsis^164,165^, chronic stress-induced depression^166^, mood disorders in general^167^ and severe TRD^168^. The effect of ketamine in reducing depressive symptoms has been shown to be fast-onset but short-lived and requires continual or maintenance treatment; however, the safety of long-term ketamine use has not yet been examined^169^. Evidence has suggested the involvement of the serotonergic and dopaminergic systems in addition to the glutamate N-methyl-D-aspartate (NMDA) receptor and BDNF^169^. Studies have postulated that excessive or ill-timed NMDA antagonism by ketamine may induce glutamate excitotoxicity, which further complicates the role of ketamine in neuroprotection or neurotoxicity, and its clinical utility^169^. Future clinical trials that examine the EPO-ketamine combination treatment would be of interest, especially in patients who have molecular measures of GSK3β given its interactions with EPO and ketamine. GSK3β cellular signalling is extremely fast acting and responsive to cellular changes. Animal studies have also shown that the monoamine reuptake inhibitor antidepressants, fluoxetine and imipramine, increase the inhibitory control of phosphorylation of Ser9 in GSK3^170,171^. Furthermore, valproate directly inhibits GSK3β and was shown to protect cells from ER stress and apoptosis^148^. Inhibition of GSK3β is therefore a possible mechanism of action shared by several classes of antidepressant medication and other emerging medications (i.e., ketamine) for the treatment of depression. Whether the pharmacological effects of these antidepressants on GSK3β contribute to the reduction of depressive symptoms is yet to be established.

## Future directions and challenges

Here we have reviewed literature that examines the relationship between EPO, mood disorders, cognition and the hippocampus. We then speculated that EPO inhibits GSK3β activity and subsequently might alter complex signalling cascades to improve cognition via hippocampal brain changes. A key limitation of this review is that the selective molecular effects of the treatment with EPO remain unclear. Also, other antidepressant therapies have strong overlap with these cellular pathways presented in this review indicating that much more work is required to unravel directly relevant versus secondary molecular events in the context of cognitive and hippocampal in mood disorders. One key area that needs prioritising is to explore cellular differentiation molecular mechanisms. Future studies investigating the effects of EPO on different cellular networks mediated by GSK3β are highly warranted to identify its common and specific roles in the treatment of mood disorders and other neuropsychiatric illnesses. Given the similarities and known differences between MDD and BD, further exploration of the underlying mechanism that differentiates unipolar and bipolar depression is necessary for novel treatment of these debilitating and chronic mood disorders. Nevertheless, the beneficial effects of EPO on cognition and hippocampal volume have been observed across several neuropsychiatric diseases including MDD, BD^40^ and schizophrenia^67^, suggesting that EPO modulates common signalling pathways involved in neuroplasticity and cognition across these disorders. Preliminary evidence suggests that GSK3β inhibition may play a role in improving a range of cognitive deficits^172,173^. We therefore recommend that further studies directly test for associations between hippocampal-related cognitive measures in mood disorders and GSK3β-related genetic networks (e.g., ITPR1) as well as considering co-treatment designs with EPO, such as ER stress inhibitors^58^. This may lead to future potential treatment options more targeted for illness-related cognitive impairments^173^.

Additional research is required to elucidate the role of the *EPO* gene (OMIM: 133170) and its related genetic variation; surprisingly, this has not been studied in mood disorders (or psychiatric disorders more generally). To our knowledge, only one study to date has investigated genetic variants across *EPO* and *EPOR* in schizophrenia, which showed initial promising results in cognitive modulation^43^. Given the caveats for genetic association studies and recruitment challenges for EPO patient studies, interactions between *GSK3β* and *EPO*/*EPOR* also require further examination using large scale, well powered healthy participant populations. In vivo work examining the functional effects of *EPO*/*EPOR* and *GSK3β* will also be an important avenue for further investigation.

The evidence to date suggests that EPO has potential clinical utility to reduce cognitive deficits in patients with depression. There is no known pharmacokinetic drug–drug interaction and no adverse events were observed in the recent EPO clinical trials^33,34^. Nevertheless, significant adverse events for EPO treatment have been reported including tumour progression and thromboembolic events. Given these potential risks of EPO treatment, extensive screening is necessary prior to starting EPO therapy and EPO-treated patients must also be closely monitored (for details, see ref. ^74^). Furthermore, the long-term benefits and use of EPO in patients need to be demonstrated in clinical studies with longer-term follow-up times regarding its potential benefits and risks. Specifically, studies using six months follow-up assessments of cognition and functioning are highly warranted given the short (6 weeks) follow-up times in the recent trials in BD and MDD. Knowing the biological and molecular genetic mechanisms, and pharmacogenetics underlying the effects of EPO, may guide clinicians and patients in understanding who will tolerate and respond to EPO treatment. This will allow clinicians to choose the best medications for each individual patient for precision medical care.

Given the highly pleiotropic effects of GSK3β in triggering multiple pathways and processes, including cancer development and tumour growth^174^, it is important to extensively investigate molecular targets that act with less potency and greater GSK3β downstream specificity (i.e., its numerous substrates). However, in vivo and in vitro evidence is currently limited and many unknown putative GSK3β substrates may exist. The identification of more GSK3β substrates is therefore of great importance for understanding the larger impact GSK3β plays in hippocampal volume of individuals with mood disorders. Algorithms that estimate the likelihood of proteins binding to GSK3β should facilitate this work^175^. Furthermore, exploration of protein kinases required for priming phosphorylation prior to GSK3β protein docking should also be explored, as is being explored in cancer research^174^. Also crucial will be harnessing the power of statistical methods, such as machine learning, to better understand how genotypic combinations combinations interact across these substrates and upstream regulating proteins as part of *GSK3β*’s larger genetic network. The identification of *GSK3β*-related genotypic combinations that specifically influence hippocampal volume in MDD may help guide further exploration for additional and more specific therapeutic combinations of treatment targets^58^.

Issues that require further exploration include disease onset and specificity. It is unclear how such factors relate to addressing issues of disease susceptibility and disease progression. While researchers have started to address complex interactions with disease onset^176^, future studies are needed, including longitudinal studies involving risk populations, to further our understanding and to further elucidate the causal pathway of antidepressant actions in the context of mood disorders. Growing evidence for dynamic neurodevelopmental patterns of GSK3β poses many additional research questions^177^. In this review, we have shown evidence that EPO may have generalizable effects spanning across illnesses; therefore, much work in this area is required to understand comorbidity and disease specificity. Furthermore, EPO neuroprotection is only partially reduced by the inhibition of pro-survival PI3K/AKT signalling^45^. In addition, GSK3β inhibition suppresses pro-inflammatory responses involving the NFκB pathway^178^; however, EPO may activate neuroinflammation signalling pathways (and other unknown molecules) using different mechanisms of action from GSK3β. Likewise, the complex relationship between EPO, GSK3β and MAPK^179^ needs further mapping. Overall, it is highly warranted to further delineate the EPO-GSK3β pathways involved in different aspects of treatment and illness.

The important role of EPO in cognition and mood disorders sheds light on the longstanding treatment challenge in psychiatric patients who suffer from chronic cognitive deficits. Unearthing the cellular pathways governed by EPO may enhance translation of targeted therapeutic strategies for mood disorders and other related conditions. Further work that explores molecular mechanisms related to the inhibition of GSK3β function and the promotion of EPO function within a mood disorder and cognition framework is highly warranted.

Restoration of neuroplasticity, including upregulation of neurogenesis and BDNF, may be an important mechanism of chronic antidepressant treatment. Mechanistically distinct compounds, such as EPO, which directly increases cellular resilience and plasticity hold great promise as novel faster acting treatments of depression. To assess the importance of such mechanisms for the cognitive and potential antidepressant effects of EPO, it would be a conceptually important next step to assess the effects of hippocampal irradiation, which impedes upregulation of BDNF and neurogenesis and blocks the behavioural effects of antidepressant drugs^180^. The associations presented in this review are only beginning to scratch the surface of the upstream and downstream events that may be changing as a result of EPO administration in relation to GSK3β and much more work is needed in this important area.
